# Correlation between plasma endothelin-1 levels and severity of septic liver failure quantified by maximal liver function capacity (LiMAx test). A prospective study

**DOI:** 10.1371/journal.pone.0178237

**Published:** 2017-05-23

**Authors:** Magnus F. Kaffarnik, Navid Ahmadi, Johan F. Lock, Tilo Wuensch, Johann Pratschke, Martin Stockmann, Maciej Malinowski

**Affiliations:** 1 Charité–Universitätsmedizin Berlin, Department of General, Visceral and Transplantation Surgery, Augustenburger Platz 1, Berlin, Germany; 2 University Hospital of Wuerzburg, Department of General-, Visceral-, Vascular- and Paediatric Surgery, Wuerzburg, Germany; 3 University Hospital of Homburg, Department of General-, Visceral-, Vascular- and Paediatric Surgery, Homburg, Germany; Azienda Ospedaliero Universitaria Careggi, ITALY

## Abstract

**Aim:**

To investigate the relationship between the degree of liver dysfunction, quantified by maximal liver function capacity (LiMAx test) and endothelin-1, TNF-α and IL-6 in septic surgical patients.

**Methods:**

28 septic patients (8 female, 20 male, age range 35–80y) were prospectively investigated on a surgical intensive care unit. Liver function, defined by LiMAx test, and measurements of plasma levels of endothelin-1, TNF-α and IL-6 were carried out within the first 24 hours after onset of septic symptoms, followed by day 2, 5 and 10. Patients were divided into 2 groups (group A: LiMAx ≥100 μg/kg/h, moderate liver dysfunction; group B: LiMAx <100 μg/kg/h, severe liver dysfunction) for analysis and investigated regarding the correlation between endothelin-1 and the severity of liver failure, quantified by LiMAx test.

**Results:**

Group B showed significant higher results for endothelin-1 than patients in group A (P = 0.01, d5; 0.02, d10). For TNF-α, group B revealed higher results than group A, with a significant difference on day 10 (P = 0.005). IL-6 showed a non-significant trend to higher results in group B. The Spearman's rank correlation coefficient revealed a significant correlation between LiMAx and endothelin-1 (-0.434; P <0.001), TNF-α (-0.515; P <0.001) and IL-6 (-0.590; P <0.001).

**Conclusions:**

Sepsis-related hepatic dysfunction is associated with elevated plasma levels of endothelin-1, TNF-α and IL-6. Low LiMAx results combined with increased endothelin-1 and TNF-α and a favourable correlation between LiMAx and cytokine values support the findings of a crucial role of Endothelin-1 and TNF-α in development of septic liver failure.

## Introduction

The liver is a key organ in the pathophysiological response to bacterial endotoxin in sepsis. Patients with septic shock develop liver failure in up to 50% of the cases and liver failure indicates poor outcome in critically ill patients [1,2]. Septic shock is accompanied by increased plasma endothelin-1 (ET-1) levels. Activation of ET-1, a proinflammatory cytokine, is associated with enhanced leukocyte trafficking, cytokine production, monocyte stimulation and profound hemodynamic changes complicating septic shock [3,4]. Particularly the regulation of liver perfusion during sepsis largely depends on ET-1-receptor activity. Experimental data indicate that bacterial endotoxin exposure leads to elevation of liver ET-1 due to release of tumor necrosis factor (TNF-α) and other inflammatory cytokines by Kupffer cells. These mediators bind to receptors on liver endothelial cells and cause increased liberation of ET-1. Elevated ET-1 provokes a strong sinusoidal vasoconstriction, which may lead to a microcirculatory impairment of liver parenchyma with heterogeneous perfusion, focal hypoxia and, finally to hepatic failure [5–8].

The reliable detection of ET-1 is limited by its instability and short plasma half-life of 1–2 minutes. Hence, an immunoluminometric assay for measurement of the c-terminal ET-1 precursor fragment (CT-proET-1) was developed. CT-proET-1 correlates well with the release of the active ET-1 and can be used to indirectly measure the release of ET-1 in physiological and pathological conditions [9,10].

Determining the severity of liver dysfunction is still a challenge due to low specificity of liver function tests [11]. Previous studies have shown that the Maximal Liver Function Capacity (LiMAx test) provides a reliable non-invasive diagnostic tool for determining the enzymatic liver function in different clinical settings [12–15].

The LiMAx test is based on the selective enzymatic demethylation of intravenously administered ^13^C-labeled methacetin by cytochrome P450 1A2 in hepatocellular cells. Our recently published data demonstrated the LiMAx test as an adequate diagnostic tool to detect early liver dysfunction in sepsis with high specificity and sensitivity [16].

The aim of this study is to investigate prospectively plasma levels of CT-proET-1, TNF-α and Interleukin-6 (IL-6) in relation to the severity of septic liver failure, measured by LiMAx test. The study also focuses on establishing the correlation between LiMAx test and proinflammatory cytokines.

## Methods

For this study, we collected and evaluated plasma samples from a patient collective of 28 septic patients admitted to the surgical intensive care unit (SICU) of the Charité, University Hospital, Berlin, Germany. The patients are the same septic patients we investigated recently for the evaluation of the LiMAx test in sepsis [16]. Patients were recruited and probes were sampled between October 2011 and February 2013. Detailed characteristics of the study population, the study design, sepsis therapy, determination of baseline parameter and parameter over the study period have been reported previously [16]. Briefly, inclusion criterion was new onset of bacterial sepsis (less than 24h) according to the 2003 International Sepsis Definitions Conference [17]. All patients also met the new criteria for sepsis according to the Third International Consensus Definitions for Sepsis and Septic Shock (Sepsis-3) [18]. Exclusion criteria were pre-existing liver diseases (e. g. cirrhosis, fibrosis, hepatitis) and liver surgery in the last 6 months.

After inclusion, LiMAx test and blood samples collection for the measurement of CT-proET-1, TNF-α and IL-6 plasma levels were performed at baseline (<24h after onset of sepsis) and on 2^nd^, 5^th^, and 10^th^ days.

The aim of the study was to establish a correlation of CT-proET-1, TNF-α and IL-6 values with the severity of liver failure, measured by LiMAx test. For this the patient population was divided into two groups: (A) Moderate liver dysfunction (LiMAx ≥100 μg/kg/h, n = 17) and (B) severe liver dysfunction (LiMAx <100 μg/kg/h, n = 11). In addition the relationship between LiMAx test, CT-proET-1, TNF-α and IL-6 was investigated.

The study was approved by the Ethics Review Board of the Charité Medical Faculty in accordance with the provisions of the Helsinki Declaration. Written informed consent was obtained from all participants or their responsible relatives prior to their participation in the study.

### Measurements of CT-proET-1, TNF-α and IL-6

In each patient, serial blood samples were drawn using an indwelling arterial catheter into EDTA tubes (EDTA-Monovette, Sarstedt, Nuernberg, Germany). All blood samples were centrifuged according to the requirement of the detection kit and the supernatant was frozen in aliquots at -80°C immediately. CT-proET-1, TNF-α and IL-6 were batch measured after completing the last patient.

Plasma CT-proET-1 levels were analyzed by a fully automated sandwich immunoassay (CT-proET-1 KRYPTOR^TM^, B.R.A.H.M.S., Hennigsdorf, Germany) [9]. For the analysis of TNF-α and IL-6 solid phase ELISA immunoassays were performed according to the instructions of the manufacturer (Quantikine human TNF-α immunoassay and Quantikine human IL-6 immunoassay, R&D systems, Inc, Minneapolis, USA). Routine laboratory parameters were measured according to the standards of the laboratory institute of the Charité—University Hospital Berlin. Normal ranges were defined for procalcitonin <0.5 μg/l, C-reactive protein <5 mg/l, white blood count 3.9–10.5 /nl, total bilirubin <1.2 mg/dl and INR 0.9–1.25 respectively.

### LiMAx test

The LiMAx test was performed using the FLIP^TM^ Analyzer (Humedics GmbH, Berlin, Germany). A bolus of 2 mg/kg body weight ^13^C-labelled methacetin was given intravenously and exhaled breath was continuously analyzed. Prior to substrate injection, the baseline ratio of ^13^CO_2_/^12^CO_2_ concentration was recorded in the native exhaled air. After metabolising in the liver, the proportion of exhaled ^13^C-labelled CO_2_ and physiological ^12^C CO_2_ (delta over base) was measured online over a period of 60 min maximum and the LiMAx value was calculated following the previously described formula [15]. In mechanical ventilated patients a special adapter was used to dissipate the exhaled gas to the ^13^CO_2_-detector. If patients were on continuous haemodialysis, the injection of ^13^C-methacetin was performed after interrupting the blood pump for 60 minutes to avoid recirculation and altered kinetics. Results are given in μg/kg/h, and the normal range is >315 μg/kg/h.

### Statistical analysis

All statistical analyses were performed using a statistical software package SPSS 19.0 of (Chicago, IL, USA). Data are presented as median value with inter-quartile range illustrated with box-and-whisker plots for non-normally distributed data. Outliers are plotted as separate dots. Individual time points were compared using repeated measures analysis of variance. Group differences were compared by the appropriate tests according to scale and data distribution, including the Mann-Whitney-U-Test and the independent t test. Correlations between quantitative variables were tested using Spearman’s rank correlation coefficient for ordinal data. Statistical significance was considered at P <0.05 and P <0.0125 respectively.

## Results

### Progression of liver function, endothelin-1 and cytokines in sepsis

Twenty-eight patients admitted to the SICU who met the inclusion criteria of sepsis, were enrolled for this study. The clinical characteristics for study inclusion are shown in Table 1.

**Table 1:** Clinical characteristics.

Number of patients	28
Age (years)	67 ± 10 (36–80)
Gender (m/w)	20/8
Septic focus	
Abdomen	15
Lung	9
Other	4
Severity of sepsis	
Sepsis	2
Severe sepsis	2
Septic shock	24
APACHE-II-score^†^	26 ± 11 (10–49)
SOFA score^†^	9 ± 5 (0–18)
SAPS-II score^†^	52 ± 19 (18–90)
Procalcitonin (μg/l)^†^	23 ± 26 (1–75)
C-reactive protein (mg/l)^†^	162 ± 84 (40–330)
WBC (/nl)^†^	15 ± 14 (1–47)
Bilirubin (mg/dl)^†^	1 ± 1 (0,2–3,7)
INR^†^	1.5 ± 0.3 (1–2)
labMELD score	18 ± 6 (8–31)

Data are presented as mean ± standard deviation (range). APACHE, Acute Physiology and Chronic Health Evaluation; SOFA, Sequential Organ Failure Assessment; SAPS, Simplified Acute Physiology Score; WBC: white blood cell count; INR International Normalised Ratio.

^†^Data collected on day 0.

**Fig 1:**
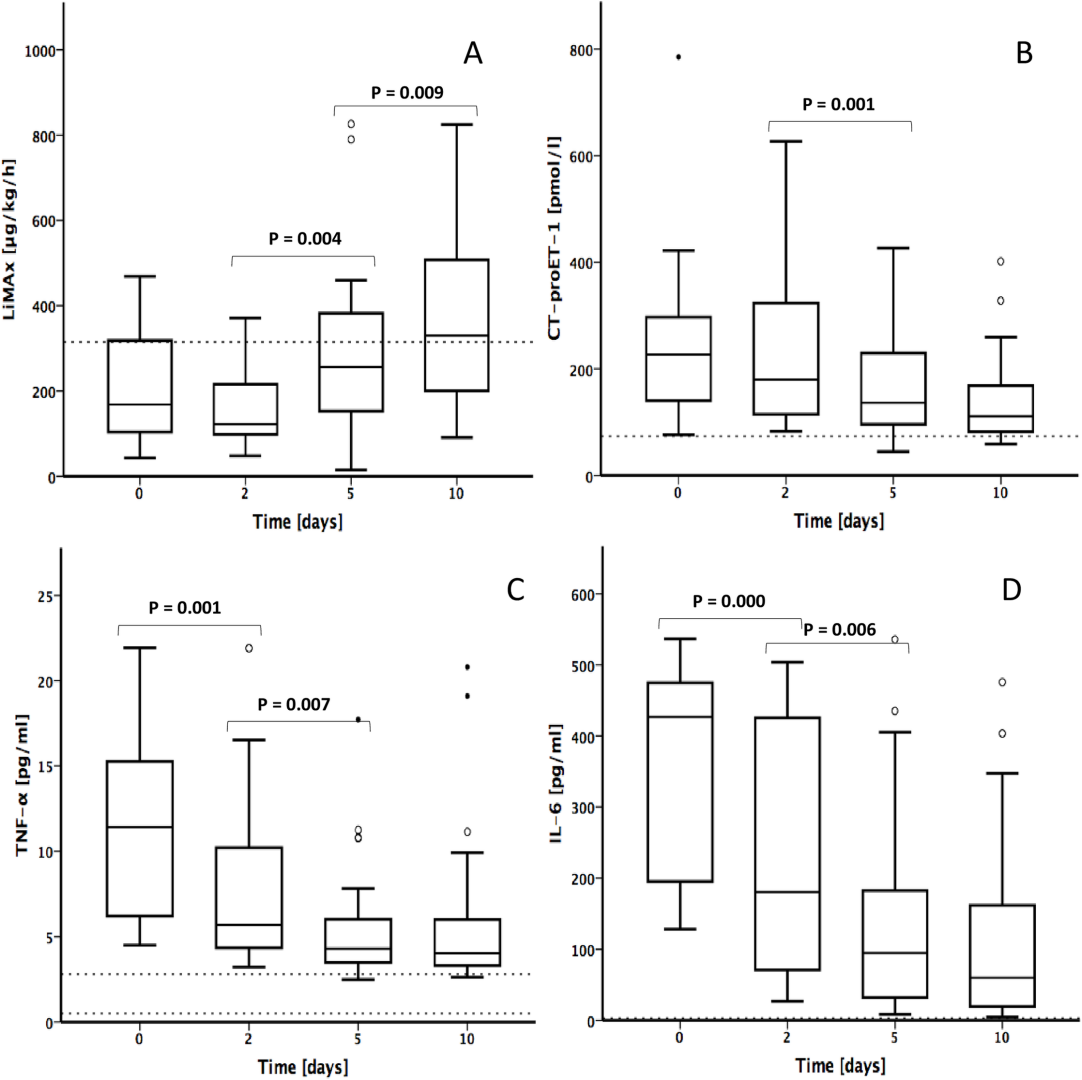
Progression of liver function, CT-proET-1 and cytokines in septic patients. **(A)** Maximal liver function capacity (LiMAx), **(B)** CT-proET-1, **(C)** TNF-α, **(D**) IL-6. Bold lines: medians; box plots: 25th to 75th percentiles. Horizontal broken lines indicate the normal range. Outliers are illustrated as circles.

LiMAx results have been reported recently in detail [16]. For better illustration we show specific LiMAx data again. In septic patients, mean baseline LiMAx values on day 0 were below normal range (208 ± 131 μg/kg/h), dropping until day 2 (165 ± 93 μg/kg/h; n. s.) and significantly increasing on day 5 (289 ± 190 μg/kg/h; P = 0.004) until day 10 (357 ± 179 μg/kg/h; P = 0.009; Fig 1A). Mean plasma levels of CT-proET-1 in septic patients were above the normal range (<74 pmol/l) with highest values on day 0 (247 ± 143 pmol/l), stepwise decreasing on day 2 (233 ± 144 pmol/l; n. s.) and day 5 (167 ± 90 pmol/l; P = 0.001) until day 10 (143 ± 86 pmol/l; n. s.; Fig 1B). Mean plasma levels of TNF-α were above the normal range (0.5–2.8 pg/ml) whilst the whole course of sepsis with a peak at day 0 (11 ± 5 pg/ml), followed by a decrease on day 2 (8 ± 5 pg/ml; P = 0.001) and day 5 (6 ± 3 pg/ml; P = 0.007), proceeding constantly until day 10 (6 ± 5 pg/ml; n. s.; Fig 1C). The IL-6 serum levels were above the normal range (<3.1 pg/ml) at each time point with highest values on day 0 (362 ± 144 pg/ml), constantly decreasing on day 2 (218 ± 165 pg/ml; P < 0.001) and day 5 (135 ± 142 pg/ml; P = 0.006) until day 10 (118 ± 135 pg/ml; n. s.; Fig 1D).

### Progression of liver function, CT-proET-1 and cytokines according to the severity of liver dysfunction

**Fig 2:**
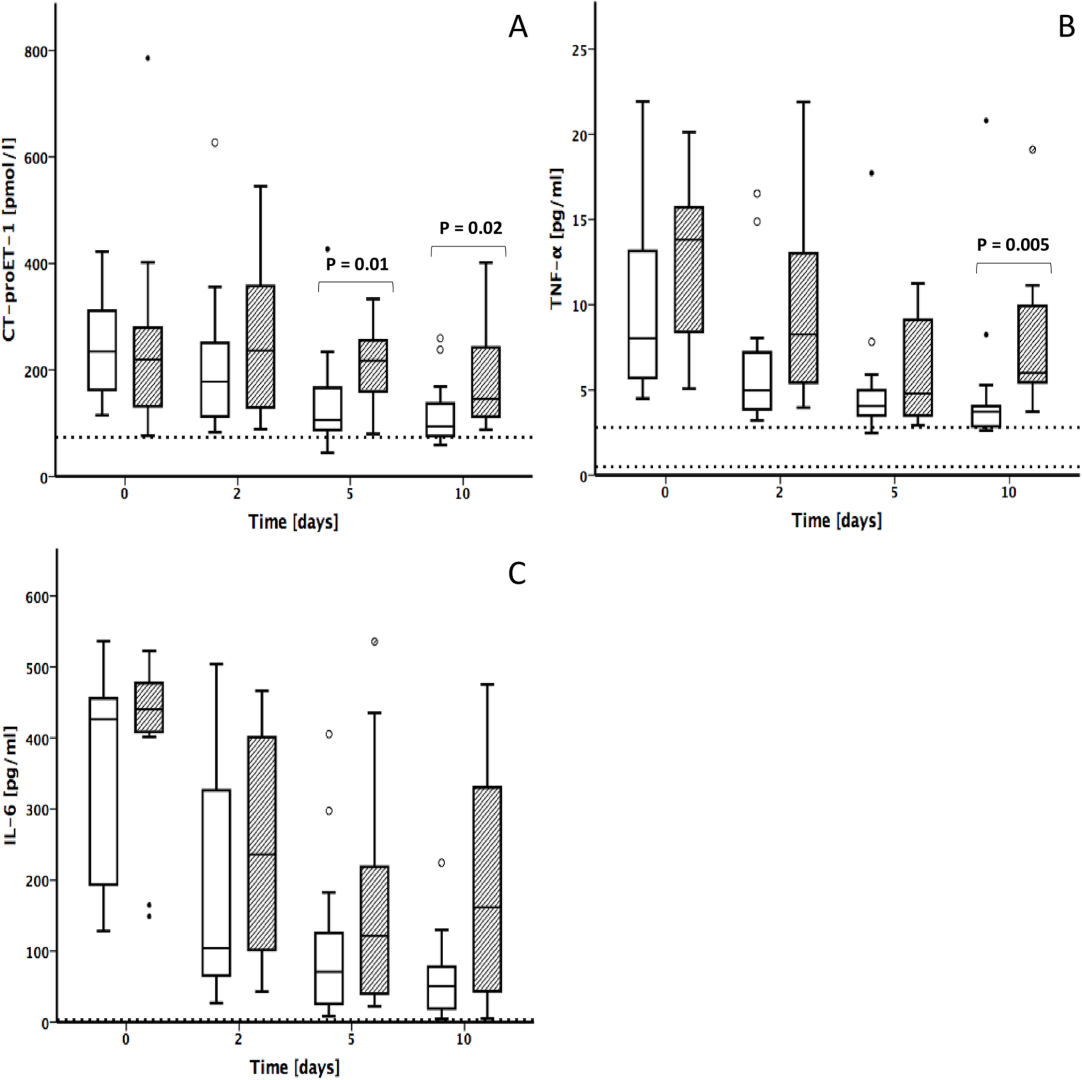
Progression of CT-proET-1 and cytokines by severity of liver dysfunction in sepsis. **(A)** CT-proET-1, **(B)** TNF-α, **(C)** IL-6. White box: group A; LiMAx ≥100μg/kg/h; Shaded box: group B; LiMAx <100μg/kg/h. Bold lines, medians; box plots, 25th to 75th percentiles. Horizontal broken lines indicate the normal range. Outliers are illustrated as circles.

Group B showed a higher severity of illness than group A expressed in significantly higher APACHE II (34.54 ± 9 vs. 21.31 ± 8.6, P = 0.002) and SOFA scores (14.54 ± 3.8 vs. 9 ± 4.1, P = 0.003). Static liver function tests also revealed higher values in patients with severe liver dysfunction (group B vs. group A: bilirubin 3.98 ± 2.9 vs. 1.64 ± 2.6, P = 0.011; INR 1.87 ± 0.3 vs. 1.58 ± 0.2, P = 0.02, Table 2). CT-proET-1 baseline values on day 0 did not differ in both study groups (group A: 239 ± 96 pmol/l; group B: 259 ± 199 pmol/l). From day 2, patients of group A showed lower values for CT-proET-1 than patients of group B. The peak of CT-proET-1 levels in group B was revealed on day 2 (264 ± 156 pmol/l), followed by a continuous decrease until day 10 (188 ± 106 pmol/l), whereas CT-proET-1 levels in group A showed the highest level on day 0 (239 ± 96 pmol/l), dropping down continuously until day 10 (116 ± 60 pmol/l). Statistical significant differences appeared on days 5 and 10 (P = 0.01 day 5; P = 0.02 day 10). Values of both groups remained above the cut off up to day 10 (Fig 2A).

**Table 2:** Clinical characteristics and severity of illness in the study groups.

	Group A	Group	
	LiMAx ≥100μg/kg/h	LiMAx <100μg/kg/h	
	n = 17	n = 11	
Age (years)	68.58 ± 11.4 (36–87)	64.78 ± 8.7 (51–77)	n. s.
APACHE-II-score^†^	21.31 ± 8.6 (10–40)	34.54 ± 9 (18–49)	P = 0.002
SOFA score^‡^	9 ± 4.1 (1–17)	14.54 ± 3.8 (7–21)	P = 0.003
SAPS-II score^‡^	54.23 ± 17.2 (27–85)	70.18 ± 21.7 (40–95)	n. s.
Procalcitonin (μg/l)^‡^	28.84 ± 27.4 (1–82)	24.47 ± 25.6 (1–73)	n. s.
C-reactive protein (mg/l)^‡^	231.74 ± 74.6 (68–349)	230.91 ± 68.3 (124–340)	n. s.
WBC (/nl)^‡^	18.33 ± 5.7 (8–29)	33.80 ± 12.9 (16–55)	P = 0.001
Bilirubin (mg/dl)^‡^	1.64 ± 2.6 (0.3–11)	3.98 ± 2.9 (0.4–19)	P = 0.011
INR^‡^	1.58 ± 0.2 (1.2–2.1)	1.87 ± 0.3 (1.4–2.4)	P = 0.020
labMELD score	16 ± 5 (27–8)	21 ± 7 (31–11)	n. s.
Mortality n (%)	0 (0)	6 (55)	P<0.001

Data are presented as mean ± standard deviation (range). APACHE, Acute Physiology and Chronic Health Evaluation; SOFA, Sequential Organ Failure Assessment; SAPS, Simplified Acute Physiology Score; WBC: white blood cell count; INR International Normalised Ratio.

^†^ Day 0.

^‡^ Highest value during study course.

Patients of group A revealed lower TNF-α values than patients of group B. Group A showed the highest levels for TNF-α on day 0 (10 ± 6 pg/ml) with a continuous decrease until day 10 (5 pg/ml ± 4). The peak in group B appeared on day 0 (13 ± 5 pg/ml), followed by a decrease until day 5 (6 ± 3 pg/ml) and another increase on day 10 (8 ± 5 pg/ml). Values on day 10 showed a statistical significant difference (P = 0.005 day 10). Values of both groups remained above the cut off (Fig 2B).

The distribution of IL-6 demonstrated a non-significant trend to higher values in patients of group B. The highest values revealed on day 0 for both groups (group A: 332 ± 153 pg/ml, group B: 403 ± 127 pg/ml). Whereas the data of group A decreased continuously until day 10 (62 ± 61 pg/ml), values of group B showed another increase on day 10 (188 ± 171 pg/ml). Values of both groups remained above the cut off up to day 10 (Fig 2C).

### Correlation between CT-pro-ET-1, TNF-α, IL-6 and liver dysfunction

The Spearman’s rank correlation test showed a statistically significant negative correlation between CT-pro-ET-1 and impaired liver function on days 2, 5 and 10 with Spearman’s rank correlation coefficients of -0.441 (day 2 + 5; P = 0.019) and -0.503 (day 10; P = 0.012). Values of TNF-α revealed a statistically significant negative correlation with the liver dysfunction on days 2 and 10 as indicated by Spearman’s coefficient of -0.537 (d2; P = 0.003) and -0.460 (d10; P = 0.027). The correlation between IL-6 serum level and liver dysfunction was statistically significant on days 2, 5 and 10 with Spearman coefficients of -0.615 (d2; P = 0.001), -0.549 (d5; P = 0.004) and -0.674 (d10; P = 0.001), respectively.

**Fig 3:**
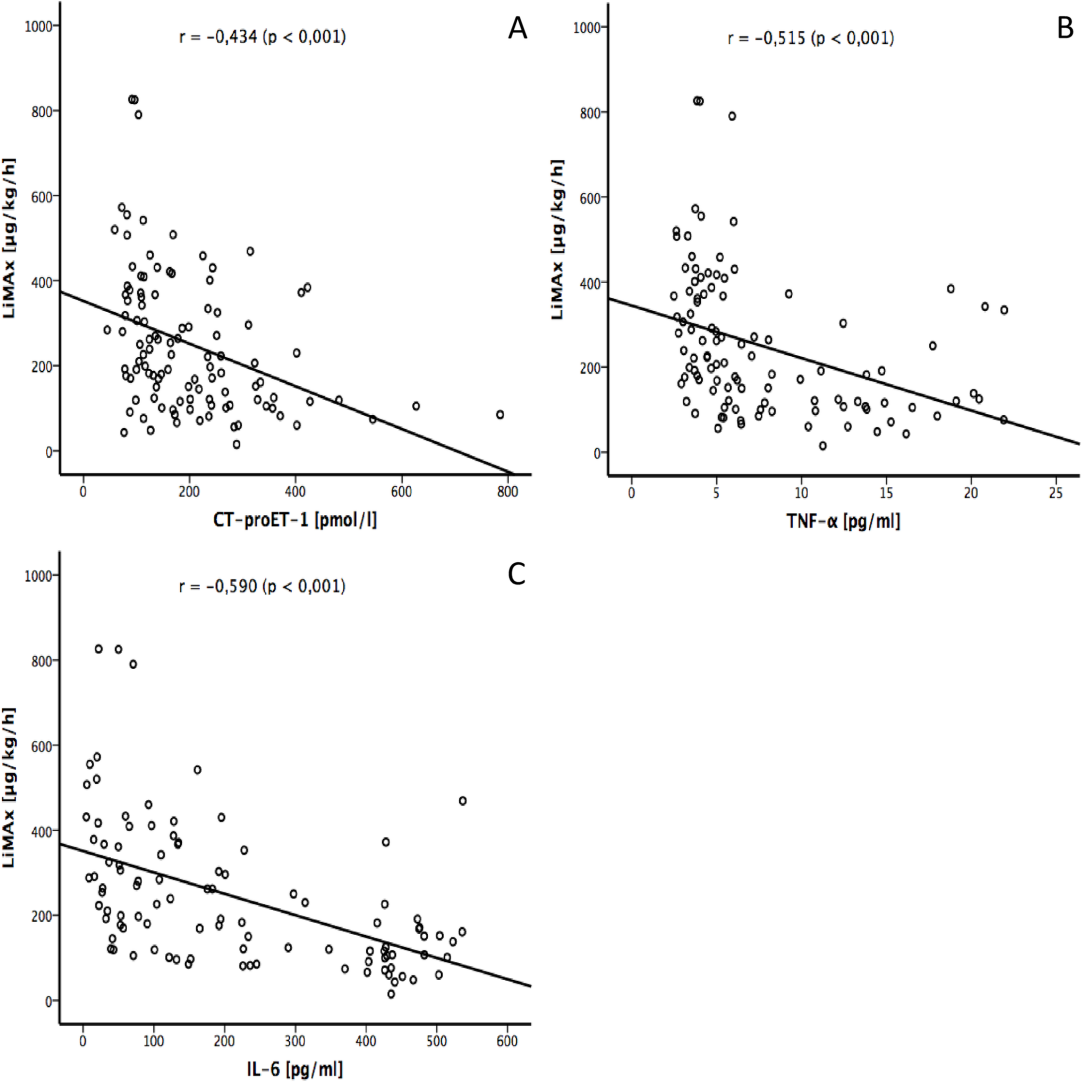
Relationship between liver function and cytokines in septic patients using Spearman’s rank correlation coefficient. **(A)** LiMAx vs. CT-proET-1, **(B)** LiMAx vs. TNF-a, **(C)** LiMAx vs. IL-6.

The overall correlation between CT-pro-ET-1, TNF-α and IL-6 and liver dysfunction showed Spearman correlation coefficients of -0.434 (CT-pro-ET-1; P <0,001, Fig 3A), -0.515 (TNF-α; P <0,001, Fig 3B) and -0.590 (IL-6; P <0,001, Fig 3C), respectively.

## Discussion

The presented prospective observational cohort study investigated the correlation between septic liver failure, the vasoactive peptide ET-1 and the proinflammatory cytokines TNF-α and IL-6 in a clinical setting. The study design, covering a period of 10 days after onset of sepsis, provided more detailed information about dynamics of the progression of the peptides as described in previous studies [19–21]. In addition to the promising results of the dynamic LiMAx test for the assessment of liver dysfunction in sepsis [16], the study shows a correlation between proinflammatory cytokines and the quantified enzymatic liver function for the first time.

The main finding of the study is the correlation between elevated CT-pro-ET-1 levels and low LiMAx values. Moreover, the negative correlation between LiMAx, CT-pro-ET-1 and TNF-α suggests an interaction between liver failure and these peptides, which leads to the conclusion that ET-1 and TNF-α may have an important impact on the development of liver failure in sepsis. These findings are in agreement with the results of Brauner and colleagues, who described elevated ET-1 levels in septic patients during a 24 hours course of data collection with higher values in non-survivors [19]. The inverse correlation between ET-1 and LiMAx is not strong (r = 0.434) but significant (P < 0.001), similar to the correlation between TNF-α and LiMAx (r = 0.515 and 0.59, P < 0.001). However, the correlation between quantitative liver function and ET-1 confirms the findings that elevated ET-1 might be one reason for decreased liver function. These findings are in accordance with previous studies reporting a pivotal impact of ET-1 in development of organ failure in sepsis due to a reduction of splanchnic, renal and pulmonary blood flow [22,23].

Experimental data indicate that regulation of sinusoidal liver perfusion strongly depends on ET-1 during sepsis [24,25]. Iwai and colleagues described a remarkable liver enzyme leakage after infusion of ET-1 in combination with endotoxin in rats. The authors concluded that ET-1 have an important role in the development of experimental liver injury related with endotoxemia and cytokine production [26]. Our results confirm these findings in a clinical setting with humans demonstrating a reasonable correlation between ET-1 and LiMAx results and significant higher ET-1 levels in patients with severe reduction of liver function. In addition, the simultaneous increase of TNF-α leads to the conclusion that there is an interaction between ET-1 and TNF-α in development of septic liver failure. These findings are in concert with other authors, describing the release of ET-1 by increased levels of TNF-α, resulting in a strong sinusoidal vasoconstriction with concomitant liver cell damage deteriorating liver dysfunction [4,24,27].

Several studies investigated sinusoidal vasodilatators in different settings of liver dysfunction. Demirci and colleagues came to the conclusion that ET-1 blocker delays onset of liver injury in diabetic rats [28]. Bahde and co-workers found decreased hepatic ischemia-related events after application of Endothelin blockers in liver transplanted rats. They came to the conclusion that Endothelin blockade improve the cell engraftment and liver repopulation [29,30]. Oki and colleagues described in an animal model an up-regulation of vascular endothelial growth factor by dual blockade of Endothelin [31]. In concert with these findings, the correlation between ET-1 and quantitative liver dysfunction, described in the presented study, implicates that enhancement of sinusoidal blood flow may improve liver dysfunction in septic patients. Further experimental and clinical studies should focus that issue.

The impact of ET-1 on organ failure in severe sepsis was investigated before [22,23]. The correlation between ET-1 and quantitative liver failure has not been previously reported. Using the LiMAx test as a dynamic diagnostic tool for quantification of liver function, the results emerging from the presented study show a correlation between the degree of liver dysfunction and ET-1. Interestingly, differences in ET-1 levels in patients with severe (group B; LiMAx <100 μg/kg/h) and moderate (group A; LiMAx ≥100 μg/kg/h) liver dysfunction are significant in the later course of sepsis. This observation cannot be answered systematically based on solid experimental or clinical data as more studies are required for analyzing ET-1 for a longer period of time. In contrast, Tschaikowsky and co-workers found higher big-ET plasma concentrations, a precursor peptide of ET-1, in non-surviving septic patients in the later course of sepsis. After initial declining, big-ET levels rose again and were significantly higher on day 28 in non-survivors compared to survivors. They came to the conclusion that the second rise may be the result of ET-induced factors of an ongoing septic process [23]. In the presented study ET-1 did not show a second rise during the study period of 10 days. But the second increase of TNF-α and IL-6 on day 10 in patients with severe liver dysfunction (group B) confirms this hypothesis. Because TNF-α works as an activator for ET-1 release, ET-1 may increase concomitantly, but only after day 10.

The present study has some important limitations. First, samples were taken from relatively small number of patients from a single institution. A larger series of patients in a multicenter trial is required to undertake further analysis. Second, the patient population was divided in only two investigation groups. Patients with moderate liver dysfunction content a variety of LiMAx results from normal range (≥315μg/kg/h) as well as reduced liver function (≥100–314μg/kg/h). To properly differentiate between different levels of hepatic dysfunction and combined ET-1 levels, larger patient groups with a more detailed clinical characterization of hepatic failure are needed. Third, due to the complex interactions of pro- and anti-inflammatory mediators, it is most likely that the relationship between ET-1, TNF-α and liver dysfunction is only one possible pathway for the development of liver failure in sepsis. In addition, we showed LiMAx results twice after publication recently (Fig 1A) [16]. This procedure was essential for better understanding the correlation of the investigated parameter. Nonetheless, our results support the interdependence of ET-1 and TNF-α and the possible impact of these peptides on liver dysfunction in sepsis and the results of the study may contribute to a better understanding of the role of ET-1 in septic liver failure and the potential clinical impact.

### Conclusions

In summary, this study focused on the relationship between septic liver dysfunction and proinflammatory peptides in sepsis. The results show a correlation between ET-1 and an impaired liver function, quantified by the LiMAx test, in critically ill patients. The findings approve the close interaction between TNF-α and ET-1 in sepsis. However, a larger sample size of patients and a larger number of cytokines would be needed to address the question of influence on septic liver failure and may strengthen the findings of this preliminary report.

## Supporting information

S1 Fig Progression of LiMAx, CT-proET-1, TNF-α and IL-6 by severity of sepsis defined by APACHE-II-score. (DOCX)

S2 Fig Progression of CT-proET-1, TNF- α und IL-6 by patient outcome (Patients survived and ICU LOS <30days vs. patients deceased or ICU LOS ≥30days). (DOCX)

S1 Table Serum concentration of CT-proET-1 [pmol/l], TNF- α [pg/ml] und Il-6 [pg/ml] in patienten with LiMAx <100μg/kg/h and ≥ 100 μg/kg/h. (DOCX)

S2 Table Progression of LiMAx, CT-proET-1, TNF-α and IL-6 by severity of sepsis defined by APACHE-II-score. (DOCX)

S3 Table Progression of CT-proET-1, TNF- α und IL-6 by patient outcome. (DOCX)

S4 Table Correlation between CT-proET-1, TNFα, IL-6 and liver dysfunction. (DOCX)
