# Supplementary material for: Correlation between plasma endothelin-1 levels and severity of septic liver failure quantified by maximal liver function capacity (LiMAx test). A prospective study
Source: PLoS One. 2017 May 23;12(5):e0178237. doi: 10.1371/journal.pone.0178237 (PMC5441649; doi:10.1371/journal.pone.0178237)
Supplement: S1 Fig — (DOCX) [file pone.0178237.s001.docx]

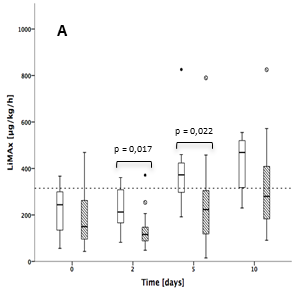


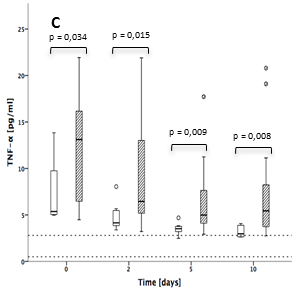


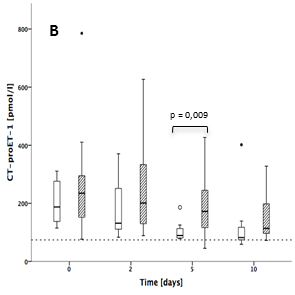


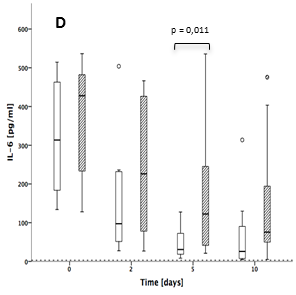


(A) LiMAx, (B) CT-proET-1, (C) TNF-α, (D) IL-6. White boxplots: APACHE-II-score <20, shaded boxplots: APACHE-II-score ≥20. Dotted line: normal range
