# Supplementary material for: Correlation between plasma endothelin-1 levels and severity of septic liver failure quantified by maximal liver function capacity (LiMAx test). A prospective study
Source: PLoS One. 2017 May 23;12(5):e0178237. doi: 10.1371/journal.pone.0178237 (PMC5441649; doi:10.1371/journal.pone.0178237)
Supplement: S2 Fig — (DOCX) [file pone.0178237.s002.docx]

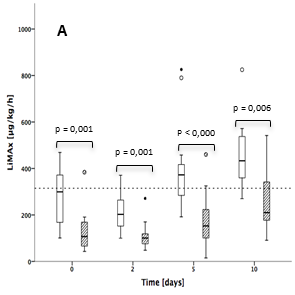


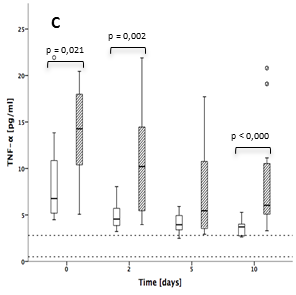


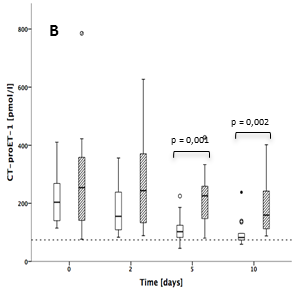


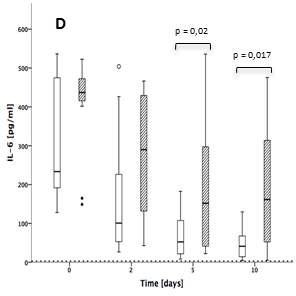


(A) LiMAx, (B) CT-proET-1, (B) TNF-α, (C) IL-6. White boxplot: Patients survived and ICU LOS <30d, shaded boxplots: Patient deceased or ICU LOS ≥30d. Dotted line: normal range
