# Supplementary material for: Correlation between plasma endothelin-1 levels and severity of septic liver failure quantified by maximal liver function capacity (LiMAx test). A prospective study
Source: PLoS One. 2017 May 23;12(5):e0178237. doi: 10.1371/journal.pone.0178237 (PMC5441649; doi:10.1371/journal.pone.0178237)
Supplement: S1 Table — (DOCX) [file pone.0178237.s003.docx]

|  | day | LiMAx <100  (n = 11) | LiMAx ≥100  (n = 17) | P value |
| --- | --- | --- | --- | --- |
| CT-proET-1 | 0 | 259 ± 199 | 239 ± 96 | n. s. |
|  | 2 | 264 ± 156 | 213 ± 137 | n. s. |
|  | 5 | 210 ± 75 | 139 ± 91 | 0.01 |
|  | 10 | 188 ± 106 | 116 ± 60 | 0.02 |
|  |  |  |  |  |
| TNF-α | 0 | 13 ± 5 | 10 ± 6 | n. s. |
|  | 2 | 10 ± 5 | 6 ± 4 | n. s. |
|  | 5 | 6 ± 3 | 5 ± 3 | n. s. |
|  | 10 | 8 ± 5 | 5 ± 4 | 0.005 |
|  |  |  |  |  |
| IL-6 | 0 | 403 ± 127 | 332 ± 153 | n. s. |
|  | 2 | 251 ± 160 | 194 ± 170 | n. s. |
|  | 5 | 174 ± 171 | 106 ± 113 | n. s. |
|  | 10 | 188 ± 171 | 62 ± 61 | n. s. |

Data are presented as mean ± standard deviation. n. s.: not significant
