# Supplementary material for: Correlation between plasma endothelin-1 levels and severity of septic liver failure quantified by maximal liver function capacity (LiMAx test). A prospective study
Source: PLoS One. 2017 May 23;12(5):e0178237. doi: 10.1371/journal.pone.0178237 (PMC5441649; doi:10.1371/journal.pone.0178237)
Supplement: S2 Table — (DOCX) [file pone.0178237.s004.docx]

|  | day | APACHE ≥20  (n = 20) | APACHE <20  (n = 8) | P value |
| --- | --- | --- | --- | --- |
| LiMAx | 0 | 193 ± 138 | 222 ± 107 | n. s. |
|  | 2 | 134 ± 78 | 227 ± 96 | 0.017 |
|  | 5 | 246 ± 180 | 400 ± 189 | 0.022 |
|  | 10 | 324 ± 191 | 427 ± 128 | n. s. |
|  |  |  |  |  |
| CT-proET-1 | 0 | 256 ± 160 | 203 ± 75 | n. s. |
|  | 2 | 254 ± 158 | 180 ± 107 | n. s. |
|  | 5 | 190 ± 96 | 104 ± 36 | 0.009 |
|  | 10 | 150 ± 75 | 126 ± 114 | n. s. |
|  |  |  |  |  |
| TNF-α | 0 | 12 ± 5 | 8 ± 3 | 0.034 |
|  | 2 | 9 ± 5 | 5 ± 1 | 0.015 |
|  | 5 | 6 ± 3 | 4 ± 1 | 0.009 |
|  | 10 | 7 ± 5 | 3 ± 1 | 0.008 |
|  |  |  |  |  |
| IL-6 | 0 | 380 ± 138 | 321 ± 159 | n. s. |
|  | 2 | 243 ± 165 | 161 ± 160 | n. s. |
|  | 5 | 173 ± 154 | 47 ± 42 | 0.011 |
|  | 10 | 140 ± 144 | 70 ± 106 | n. s. |

Data are presented as mean ± standard deviation. n. s.: not significant
