# Supplementary material for: Correlation between plasma endothelin-1 levels and severity of septic liver failure quantified by maximal liver function capacity (LiMAx test). A prospective study
Source: PLoS One. 2017 May 23;12(5):e0178237. doi: 10.1371/journal.pone.0178237 (PMC5441649; doi:10.1371/journal.pone.0178237)
Supplement: S3 Table — (DOCX) [file pone.0178237.s005.docx]

|  | day | patients deceased or ICU LOS ≥30 days  (n = 14) | patients survived and ICU LOS <30 days  (n = 14) | P value |
| --- | --- | --- | --- | --- |
| CT-proET-1 | 0 | 277 ± 182 | 217 ± 85 | n. s. |
|  | 2 | 283 ± 171 | 182 ± 93 | n. s. |
|  | 5 | 220 ± 93 | 115 ± 48 | 0.001 |
|  | 10 | 186 ± 97 | 101 ± 47 | 0.002 |
|  |  |  |  |  |
| TNF-α | 0 | 14 ± 5 | 9 ± 5 | 0.021 |
|  | 2 | 11 ± 5 | 5 ± 1 | 0.002 |
|  | 5 | 7 ± 4 | 4 ± 1 | n. s. |
|  | 10 | 9 ± 6 | 3 ± 1 | 0.000 |
|  |  |  |  |  |
| IL-6 | 0 | 407 ± 116 | 317 ± 159 | n. s. |
|  | 2 | 278 ± 161 | 157 ± 151 | n. s. |
|  | 5 | 201 ± 171 | 68 ± 55 | 0.020 |
|  | 10 | 185 ± 156 | 44 ± 37 | 0.017 |

Data are presented as mean ± standard deviation. n. s.: not significant
