# Supplementary material for: Correlation between plasma endothelin-1 levels and severity of septic liver failure quantified by maximal liver function capacity (LiMAx test). A prospective study
Source: PLoS One. 2017 May 23;12(5):e0178237. doi: 10.1371/journal.pone.0178237 (PMC5441649; doi:10.1371/journal.pone.0178237)
Supplement: S4 Table — (DOCX) [file pone.0178237.s006.docx]

|  |  | **LiMAx test** | | | |
| --- | --- | --- | --- | --- | --- |
|  |  | day 0 | day 2 | day 5 | day 10 |
| **CT-pro-ET-1** | day 0 | X |  |  |  |
|  | day 2 |  | -0.441 (P=0.019) |  |  |
|  | day 5 |  |  | -0.441 (P=0.019) |  |
|  | day 10 |  |  |  | -0.503 (P=0.012) |
| **TNFα** | day 0 | X |  |  |  |
|  | day 2 |  | -0.537 (P=0.003) |  |  |
|  | day 5 |  |  | X |  |
|  | day 10 |  |  |  | -0.460 (P=0.027) |
| **IL-6** | day 0 | X |  |  |  |
|  | day 2 |  | -0.615 (P=0.001) |  |  |
|  | day 5 |  |  | -0.549 (P=0.004) |  |
|  | day 10 |  |  |  | -0.674 (P=0.001) |

Nonparametric measurement of statistical dependence between two variables using Spearman's rank correlation coefficient. X = not significant.
